# When a 520 million-year-old Chengjiang fossil meets a modern micro-CT – a case study

**DOI:** 10.1038/srep12802

**Published:** 2015-08-04

**Authors:** Yu Liu, Gerhard Scholtz, Xianguang Hou

**Affiliations:** 1 Yunnan Key Laboratory for Palaeobiology, Yunnan University, Kunming 650091, People’s Republic of China; 2 Department Biology II, Biocenter, Ludwig-Maximilians-Universität München, Großhaderner Str. 2, 82152 Planegg-Martinsried, Germany; 3 GeoBio-Center LMU, Richard-Wagner-Straße 10, Munich, 80333, Germany; 4 Institut für Biologie/Vergleichende Zoologie, Humboldt-Universität zu Berlin, Philippstr. 13, 10115 Berlin, Germany

## Abstract

The 520 million-year-old Chengjiang biota of China (UNESCO World Heritage) presents the earliest known evidence of the so-called Cambrian Explosion. Studies, however, have mainly been limited to the information exposed on the surface of the slabs. Thus far, structures preserved inside the slabs were accessed by careful removal of the matrix, in many cases with the unfortunate sacrifice of some “less important” structures, which destroys elements of exceptionally preserved specimens. Here, we show for the first time that microtomography (micro-CT) can reveal structures situated inside a Chengjiang fossil slab without causing any damage. In the present study a trilobitomorph arthropod (*Xandarella spectaculum*) can be reliably identified only with the application of micro-CT. We propose that this technique is an important tool for studying three-dimensionally preserved Chengjiang fossils and, most likely, also those from other biota with a comparable type of preservation, specifically similar iron concentrations.

In 2012, a 520 million-year-old fossil site located in southwest China, the Chengjiang biota, was approved to be one of the UNESCO World Heritage sites^1^. This biota documents the earliest known evidence of the so-called Cambrian Explosion – a significant bioradiation event in which the oldest known representatives of the major animal groups known from today appeared within a relatively short geological time window^2^,^3^. Chengjiang animals, especially those with soft bodies such as sponges, ctenophores, cnidarians, and cycloneuralians were often compressed during sediment compaction, forming 2D fossils^2^. By contrast, animals that bear mineralized exoskeletons such as arthropods were often preserved in a more 3D manner, compressed but still preserving some 3D details. In particular, depending on the orientation of the animal certain structures, such as appendages, are often found a few millimeters below or above the body in the fossil slab. This is due to the resistance of the hard skeletal parts to the compression of the animals during fossilization. Until now, such hidden structures were usually accessed by careful removal of the fossil matrix, sometimes including some “less important” structures such as parts of the exoskeleton^2^. This method requires well-trained, highly capable hands and adequate investment of time. Further, such a preparation is an inherently destructive technique that causes damage to the precious fossil specimens. Studies on Chengjiang fossils, therefore, have been largely restricted to surface observation, with either traditional light microscopy (LM) or the more recent application of fluorescence microscopy (FM)^4^,^5^.

Microtomography (micro-CT) is the most commonly employed technique for 3D characterization in palaeontology^6^,^7^. During each scan the micro-CT obtains a series of X-ray radiographs (also called projections) from a specimen being rotated 360°^7^,^8^. In studies of fossil arthropods, for instance, this technique has been shown to be a powerful tool for investigating specimens preserved in ambers^9^,^10^,^11^ or siderite nodules^12^,^13^,^14^,^15^,^16^. In these cases, the technique helped researchers not only in visualizing structures that are unseen from the outside^16^, but also in creating virtual 3D models of the fossilized animals with great detail^9^,^10^,^14^,^15^. Micro-CT has not yet been widely applied on studies of Chengjiang fossils. Thus far, Tanaka and colleagues provided the only published account, in which the general profile of a putative central nervous system on one specimen of the arthropod, *Alalcomenaeus* sp., was visualized without paying much attention to 3D aspects^17^.

Here, we report the first in-depth micro-CT study on a 3D preserved Chengjiang fossil, in the sense of making use of X-rays to penetrate the fossil slab and reveal detailed structures preserved inside the slab. In this study, the structures hidden inside the slab are particularly important for tentatively identifying the superficially shrimp-like specimen as the arthropod *Xandarella spectaculum* – a rare trilobite-like species that is unique to Chengjiang^2^. Therefore, we propose that micro-CT is a powerful tool to study 3D fossils from Chengjiang and probably also those from other biota with a similar type of preservation, specifically similar iron concentrations.

## Results

### Observations with LM and FM

**Figure 1:**
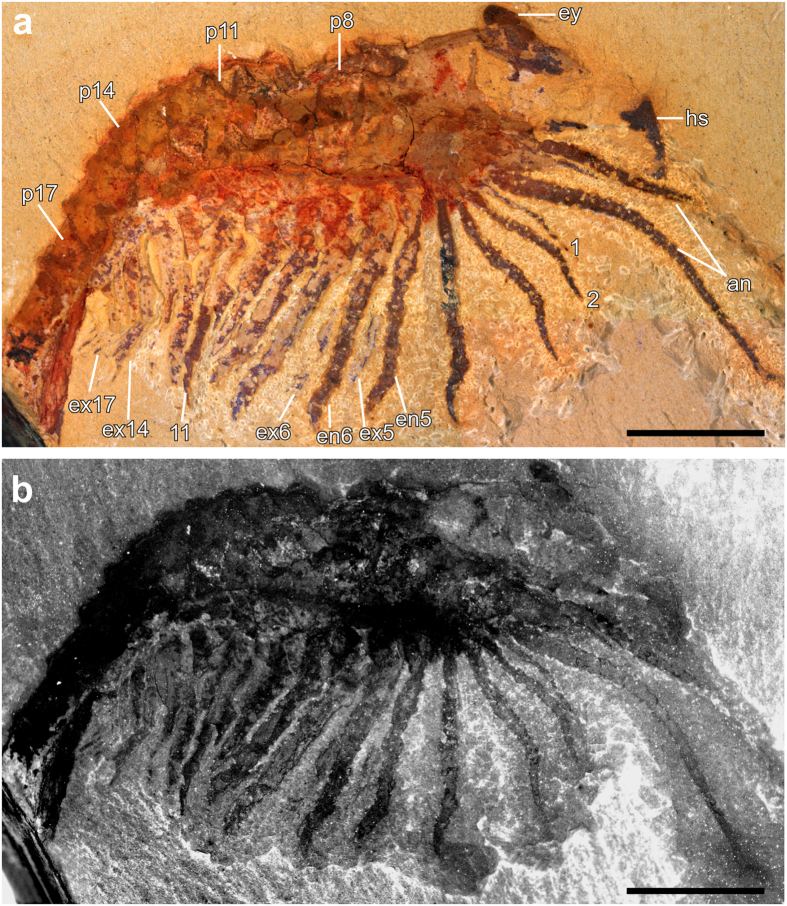
Overview of the specimen (YKLP 11086). (**a**) Light microscopy (macrophotography); (**b**) Fluorescence microscopy. Abbreviations: an, antenna; en, endopod; ex, exopod; ey, eye; hs, head shield; p8–17, post-antennal segments; en/ex1–17, endopod/exopod of post-antennal appendages. Overall length of the specimen from head to tail 24 mm, with antennae 32 mm. Scale bars, 5 mm. Photographs in **a**, **b** taken by Y.L.

As revealed with LM (Fig. 1a) and FM (Fig. 1b), the fossil arthropod appears similar to a shrimp from a lateral perspective. The anterior body part shows a head shield-like structure and a dorsal eye-like spot. The head shield seems dorsally folded. Dorsal segmentation of the body can be seen only in the posterior half. At the anterior end, the arthropod carries a pair of thick and multi-annulated antennae at the ventral side that are followed by a series of appendages. The 1^st^ post-antennal appendage is observed as uniramous, slender, and endopod-like. It is distinctly shorter and thinner than the subsequent ones (Fig. 1). The 2^nd^ to 4^th^ post-antennal appendages appear to be uniramous with LM and FM, while the 5^th^ and 6^th^ apparently carry an endopod and an exopod (Fig. 1). In the more posterior ones, starting from about the 14^th^, the setae on the exopods are seen clearly, slightly better shown under the FM than the LM (cf. Fig. 1a,b).

### Observations with micro-CT

**Figure 2:**
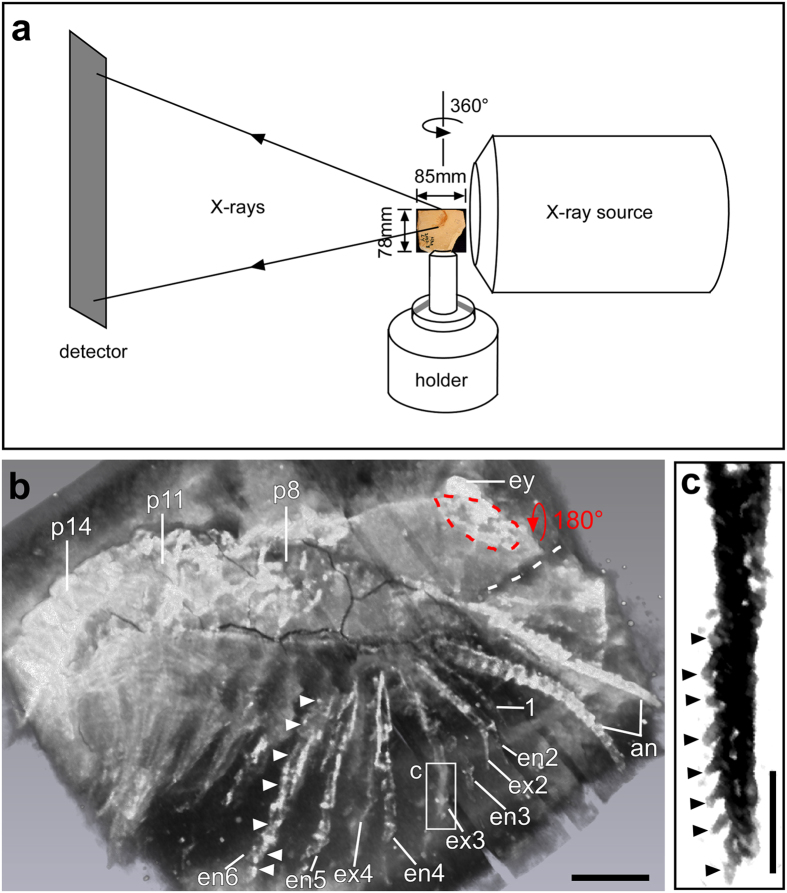
Micro-CT set-up and fine details revealed on the surface of the slab. (**a**) The slab was placed as close to the X-ray source as possible to obtain the highest possible resolution in the final micro-CT images. The slab was rotated 360° during the process of scan, with a rotation radius of 42.5 mm. In this study, the anterior (Figs 2b and 3) and posterior (Fig. 4) parts of the specimen were scanned separately. (**b**) Fine details in the anterior part of the specimen revealed with micro-CT (volume rendering, Amira). Annulation of the antennae (an) is evident. The first post-antennal appendage (1) is uniramous and appears annulated. Exopod (ex) and endopod (en) of the 2^nd^ to 4^th^ post-antennal appendages are now clearly displayed. Elongated podomeres (white arrowheads) in the endopod of the 5^th^ and 6^th^ post-antennal appendages are shown. The blue dashed line marks a small region of the head shield, which was preserved in a flipped-over manner (blue arrow). The white dashed line indicates that the two parts of the head shield were torn apart from each other. (**c**) Close-up (Hires mode in Drishti) of the exopod shows a series of setae (arrowheads) at the distal end of the 3^rd^ post-antennal appendage. Each seta is around 100 μm in length. Abbreviations as in Fig. 1. Scale bars, 2 mm in **b**, 1 mm in **c**. Photographs in **a, b** taken by G.S., in **c** by Y.L.

**Figure 3:**
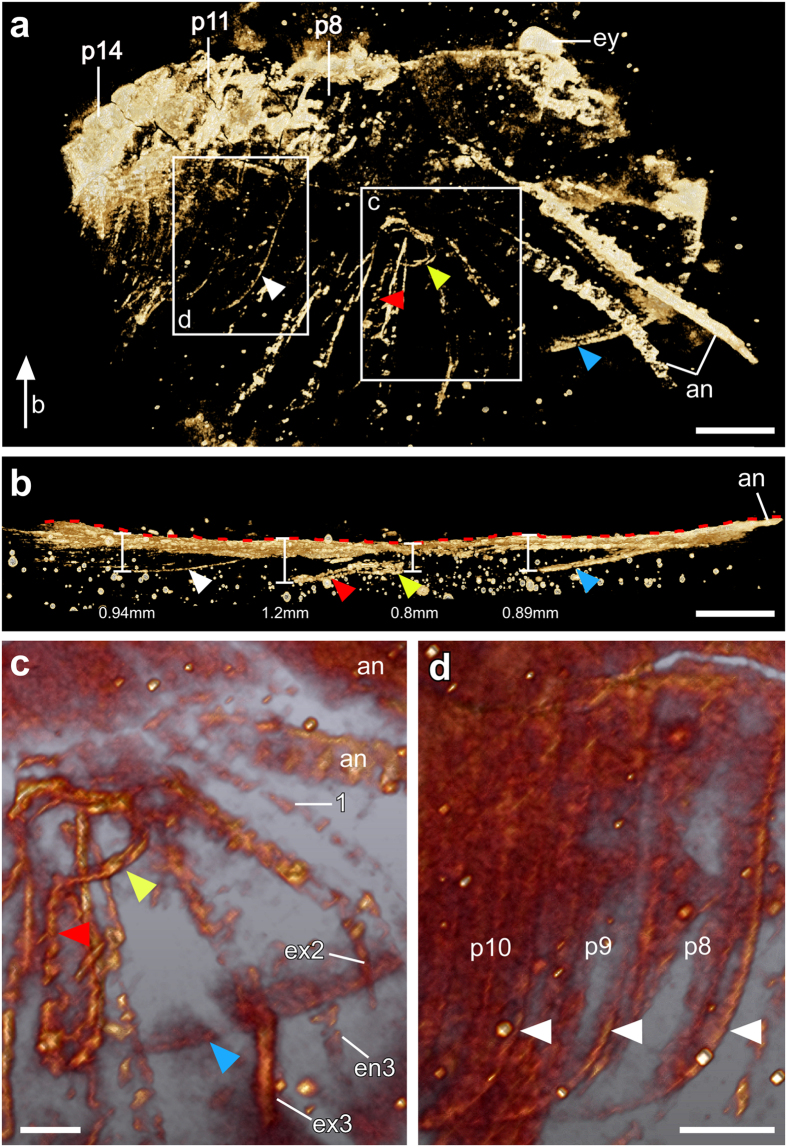
Fine details revealed inside the slab. (**a**) Overview of the main part of the specimen. Micro-CT (Hires mode, i.e. high-resolution mode, Drishti) reveals the lateral margin (blue arrowhead) of the head shield. The annulation of the antennae is more clearly shown here than with light or fluorescence microscopy (cf. Fig. 1). The pleura of several consecutive segments (white arrowhead), and an opening (yellow arrowhead) and a fissure (red arrowhead) in the head shield were revealed. The white arrow indicates the angle from **b**. (**b**) 90°-rotated anterior part of the specimen shown in **a** (Hires mode, Drishti). The red dashed line indicates the surface of the specimen. The greatest depth of the lateral margin of the head shield (blue arrowhead) is 0.89 mm, that of the opening (yellow arrowhead) is 0.8 mm, the fissure (red arrowhead) 1.2 mm, and the pleurae (white arrowhead) 0.94 mm. (**c**) Close-up (volume rendering, Amira) of one side of the head shield from **a** showing an opening (yellow arrowhead) with a fissure (red arrowhead) extending towards the lateral margin (blue arrowhead) of the head shield; (**d**) Close-up (volume rendering, Amira) of the eighth to tenth post-antennal segments (p8–p10) indicated by their respective pleura (white arrowheads). Abbreviations as in Fig. 1. Scale bars, 2 mm. Photographs in in **a, b** taken by Y.L. and in **c, d** taken by G.S.

**Figure 4:**
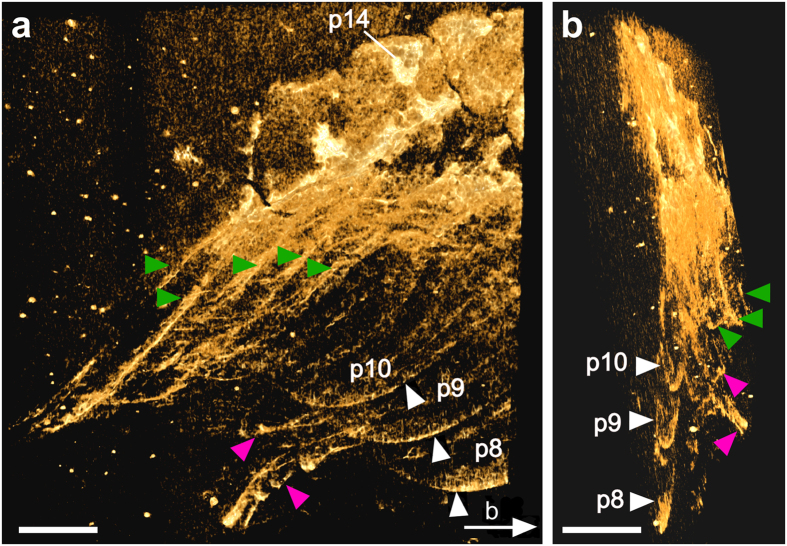
Fine details revealed in the posterior part of the specimen. (**a**) Overview of the posterior part of the specimen. Micro-CT (Hires mode, Drishti) reveals pleurae from the right (green arrowheads) and left (white arrowheads; p8–p10) sides of the body. Two appendages (magenta arrowheads) are also visible. The white arrow indicates the viewing angle from **b**. (**b**) Posterior view of the posterior part of the specimen shown in **a**. The two appendages (magenta arrowheads) are located between the right and left (p8–p10) pleurae. While the right pleurae were preserved on the surface of the slab, the appendages (magenta arrowheads) and the left pleurae of the 9th to 11th post-antennal segments (p8–p10) were inside the slab. Abbreviations as in Fig. 1. Scale bars, 2 mm. Photographs in **a**, **b** taken by Y.L.

When scanned with micro-CT (Fig. 2a; see also ‘Methods’), many additional fine details were uncovered (Figs 2, 3, 4). On the surface of the fossil slab, the multi-annulation of the antennae is now evident, and the eye spot stands out from the margin of the head shield (Figs 2b and 3a). Next to the eye spot and the frontal margin of the head shield is a small fragmentary region, most likely representing a ventrally folded margin of the head shield (Fig. 2b). The white dashed line in Fig. 2b suggests that the two parts of the head shield were torn apart from each other. The annulated structure of the 1^st^ post-antennal appendage and a second branch for each of the biramous 2^nd^ to 4^th^ post-antennal appendages can now be more clearly seen (Fig. 2b). Further, a series of setae-like armatures, each around 100 μm in length, are now visible at the distal part of the exopod of the 3^rd^ post-antennal appendage (Fig. 2c). The 8^th^ post-antennal segment is clearly separated from the posterior margin of the head shield (Figs 2b and 3a). In the posterior region of the specimen, the pleurae of about 9 consecutive segments are identified (Fig. 4).

Micro-CT revealed several structures invisible from the surface of the slab (Figs 3 and 4). In the anterior part of the specimen, these include the lateral margin of the head shield (Fig. 3a–c), the pleurae of several consecutive trunk segments (Fig. 3a,b,d), and most importantly, an opening on one side of the head shield extending into a fissure towards the margin of the head shield (Fig. 3a–c). In the rotated view (Fig. 3b), we measured the greatest depth from the surface of the slab to the lateral margin of the head shield to be 0.89 mm, the pleurae 0.94 mm, the opening 0.8 mm, and the fissure 1.2 mm. In the posterior part of the specimen, two additional appendages that were invisible from the surface were observed between the pleurae from both body sides (Fig. 4).

## Discussion

### Identification of the specimen

It has been difficult, if not impossible, to identify the specimen (YKLP 11086) solely based on the limited information exposed on the surface of the fossil slab (see ‘Results’). Neither LM (Fig. 1a) nor FM (Fig. 1b) revealed enough information to lead to a reliable identification. With micro-CT we observed three key features preserved inside the slab that enabled us to interpret the specimen as most likely a representative of *Xandarella spectaculum*. First, it has an eye opening on one side of the head shield (Fig. 3a) – a feature that is unique for *X. spectaculum*^18^. Compared to the ventrally located eyes of other Cambrian arthropods, those of *X. spectaculum* are in a more dorsal position and uniquely look up through the two openings in the dorsal head shield cuticle^2^. Second, a fissure extending from the opening towards the lateral margin of the head shield (Fig. 3a), which is another unique feature of *X. spectaculum*. On dorso-ventrally compressed specimens, the fissures on each side of the head shield connect with each other between the eyes^18^ and possibly represent an unfused segmental boundary^19^. Further, this boundary was suggested to mark the path of the eye moving from a more ventral to a more dorsal location^20^. Third, the shape of the pleurae of the trunk segments (Fig. 3a,d) is identical to previous descriptions of *X. spectaculum*^2^,^8^,^20^. Further, based on the micro-CT analysis we can identify some structures that have been ambiguous in previous descriptions of *X. spectaculum*. In particular, the 1^st^ post-antennal appendage is uniramous and most likely annulated (Fig. 2b). In contrast, the more posterior appendages are biramous and show a lower number (7) but longer cylindrical podomeres in the endopod than previously thought (Fig. 2b)^18^.

### Taphonomy of the specimen

Due to the trilobite-like body shape, until now all known specimens of *X. spectaculum* were preserved in a “regular” dorso-ventrally compressed manner^2^,^18^,^19^,^20^,^21^,^22^. By contrast, the specimen studied here documents an “irregular” orientation. As shown in Fig. 3b, the lateral margin of the head shield, the eye opening and fissure, and the pleurae of several trunk segments are preserved inside the slab (Fig. 3b). The fact that these structures are all from the left body side of the animal indicates that the animal was lying on its left side on the sediment before the fossilization started. However, the presence of two antennae, i.e. ventral perspective (Figs 2b and 3a) and the co-existence of the pleurae from both sides in the most posterior part of the specimen, i.e. lateral perspective (Fig. 4) suggest that the body of the animal was strongly twisted. In other words, we are observing the animal’s anterior part ventrally, middle part ventro-laterally, and posterior part laterally. Moreover, it appears that most post-antennal appendages and pleurae on the right side of the animal are missing on the specimen (Figs 2b and 3a).

### Potential of micro-CT in studying fossils with a similar type of preservation

In the present study, micro-CT reveals fine details preserved on the surface of the slab. These include the articulation of the antennae and putatively of the first post-antennal appendage (Fig. 2b), and the 100 μm-long setae-like armature at the distal part of the exopod of the 3^rd^ post-antennal appendage (Fig. 2c). Most importantly, the X-ray beam of the micro-CT is shown here to be able to penetrate through a 2-cm thick Chengjiang slab in which additional layers of the fossilized animal are preserved (Figs 2, 3, 4). As with most other fossils from Chengjiang^23^, the specimen studied here is deeply weathered. Compared to the more yellowish matrix, the redish/brownish surface of the animal indicates the presence of an iron-rich aluminosilicate^23^. Additionally, pyritization of nonmineralized tissues has been suggested as the principal mode of preservation for Chengjiang fossils^24^. We, therefore, speculate that it is both the iron-rich aluminosilicate and pyrite that underlie the different X-ray absorption levels between the fossilized animal and the matrix, which in turn allows the high contrast of the fossilized animal against the matrix.

Based on the above case study, we propose that the application of micro-CT can be extended into studies on fossil animals from other biota with similar type of mineral replacement. These include the recently discovered Lower Cambrian Xiazhuang fossil assemblage^25^ in Kunming and the new assemblage from the Burgess Shale of the Canadian Rockies^26^. Further, we propose that micro-CT should also be used to analyze previously published specimens from Chengjiang and similar biota. Investigations into the structures that are hidden inside the fossil slabs, such as nonbiomineralized soft tissue^27^, will allow us to achieve a more reliable descriptive basis of Cambrian body organizations and thus an improved understanding of the evolution of animal morphology.

## Methods

### Material

The investigated specimen of *Xandarella spectaculum* (YKLP 11086) is housed at the Yunnan Key Laboratory for Palaeobiology, Yunnan University, Kunming, China. The slab size is approximately 85 mm high, 75 mm wide, and 20 mm thick.

### Locality and horizon

The specimen was collected from the Yu’anshan Member of the Lower Cambrian Chiungchussu Formation (Cambrian Series 2, Stage 3) at Mafang village, Haikou, Yunnan Province, China.

### Imaging

Figure 1a was captured with a MP-E 65 mm macro objective mounted to a Canon EOS Rebel T3i digital camera, and was processed in Adobe Photoshop Elements 4.0. Figure 1b was originally documented manually as a stack of fluorescence images with a Leica DFC340 FX monochrome digital camera attached to a Leica M205 FA fluorescence stereo microscope (green-orange fluorescence). These images were fused into a sharp image in CombineZM, which was then processed in Adobe Photoshop Elements 4.0. In contrast to the surrounding matrix the specimen itself shows no fluorescence, which is characteristic of Chengjiang fossils^4^. Such inverse fluorescence has revealed fine details that are invisible with a light microscope^5^.

Images in Figs 2b,c, 3 and 4 were derived from micro-CT scans. The whole specimen was subjected to micro-tomographic analysis at the Museum für Naturkunde, Berlin, using a Phoenix nanotom X-ray tube at 90 kV and 120 μA, generating 1500 projections per scan. Effective voxel size was about 11 μm. A Cu-filter (0.3 mm) was used to cut out low energy X-rays from the source. The cone beam reconstruction was performed using the datos|x- reconstruction 2.1 software (GE Sensing & Inspection Technologies GmbH phoenix|x-ray) and the data were visualized in VG Studio Max 2.0. The micro-CT scanner used allows a maximum object size of approximately 10 cm diameter and 10 to 15 cm height. For larger slabs there are larger models available. The investigated specimen underwent no special treatment, such as cutting off parts of the slab, and was not affected by the scan. It was mounted vertically on a rotating holder with a plastic tube equipped with a slit in which the slab was inserted. In addition, the object was protected by plastic foil which was fixed with tape that did not touch the rock. The analysis of the image stacks was done with the 3D software Drishti using its hires mode (high-resolution mode)^28^ (Figs 2c, 3a,b and 4), and Amira 5.4.3 using a combination of the ortho-slice and volren modes (Figs 2b, 3c and 3d). With the ortho-slice mode the level of interest in the slab was chosen followed by volume rendering with the volren mode. Levels on top or beneath the chosen slice were removed with the clip function.

## Additional Information

**How to cite this article**: Liu, Y. *et al.* When a 520 million-year-old Chengjiang fossil meets a modern micro-CT – a case study. *Sci. Rep.* **5**, 12802; doi: 10.1038/srep12802 (2015).
